# Mitochondrial PAK6 inhibits prostate cancer cell apoptosis *via* the PAK6-SIRT4-ANT2 complex

**DOI:** 10.7150/thno.42874

**Published:** 2020-02-03

**Authors:** Tingting Li, Yang Li, Tong Liu, Bingtao Hu, Jiabin Li, Chen Liu, Tao Liu, Feng Li

**Affiliations:** 1 Department of Cell Biology, Key Laboratory of Cell Biology, National Health Commission of the PRC, and Key Laboratory of Medical Cell Biology, Ministry of Education of the PRC, Shenyang 110122, Liaoning, China.; 2 Medical Research Center, Peking University Third Hospital, 49 North Garden Road, Haidian District, Beijing 100191, China.; 3 Department of Urology, the First Affiliated Hospital of China Medical University, Shenyang 110001, Liaoning, China.

**Keywords:** PAK6, SIRT4, ANT2, apoptosis, prostate cancer.

## Abstract

**Rationale**: P21-activated kinase 6 (PAK6) is a member of the class II PAKs family, which is a conserved family of serine/threonine kinases. Although the effects of PAK6 on many malignancies, especially in prostate cancer, have been studied for a long time, the role of PAK6 in mitochondria remains unknown.

**Methods**: The expression of PAK6, SIRT4 and ANT2 in prostate cancer and adjacent non-tumor tissues was detected by immunohistochemistry. Immunofuorescence and immunoelectron microscopy were used to determine the subcellular localization of PAK6. Immunoprecipitation, immunofuorescence and ubiquitination assays were performed to determine how PAK6 regulates SIRT4, how SIRT4 regulates ANT2, and how PAK6 regulates ANT2. Flow cytometry detection and xenograft models were used to evaluate the impact of ANT2 mutant expression on the prostate cancer cell cycle and apoptosis regulation.

**Results**: The present study revealed that the PAK6-SIRT4-ANT2 complex is involved in mitochondrial apoptosis in prostate cancer cells. It was found that PAK6 is mainly located in the mitochondrial inner membrane, in which PAK6 promotes SIRT4 ubiquitin-mediated proteolysis. Furthermore, SIRT4 deprives the ANT2 acetylation at K105 to promote its ubiquitination degradation. Hence, PAK6 adjusts the acetylation level of ANT2 through the PAK6-SIRT4-ANT2 pathway, in order to regulate the stability of ANT2. Meanwhile, PAK6 directly phosphorylates ANT2 atT107 to inhibit the apoptosis of prostate cancer cells. Therefore, the phosphorylation and deacetylation modifications of ANT2 are mutually regulated, leading to tumor growth *in vivo*. Consistently, these clinical prostate cancer tissue evaluations reveal that PAK6 is positively correlated with ANT2 expression, but negatively correlated with SIRT4.

**Conclusion**: These present findings suggest the pivotal role of the PAK6-SIRT4-ANT2 complex in the apoptosis of prostate cancer. This complex could be a potential biomarker for the treatment and prognosis of prostate cancer.

## Introduction

Prostate cancer is one of the most common malignant tumors of the male urinary system. It is also one of the malignant tumors with a rapid increase in incidence 1. Astonishingly, new diagnoses of prostate cancer alone will account for one-fifth of cancers in men by 2019 2. At present, endocrine therapy, radiotherapy and chemotherapy are still used in clinic, and these still leads to the recurrence and metastasis of prostate cancer, leading to death 1, 3-5, and the underlying molecular mechanism remains incompletely characterized.

P21-activated kinase 6 (PAK6) is a member of the class II PAKs family, which is a conserved family of serine/threonine kinases 6, 7. The oncogene is overexpressed in a huge variety of human cancers 8-13, including prostate cancer 14-19. In addition, studies have shown that PAK6 is positively correlated with the malignant degree of prostate cancer and preoperative prostate-specific antigen (PSA) levels.

Furthermore, PAK6 is especially amplified in prostate cancer, with recurrence after castration treatment 8, 17. The overexpression and maintenance of PAK6 kinase activity can reduce the sensitivity of tumor cells to radiotherapy and chemotherapy, leading to the recurrence of prostate cancer 14, 15, 20. The cellular localization of PAK6 varies with the cell type and environment. For example, in prostate cancer LNCaP cells, PAK6 localizes in the nucleus 16, thereby inhibiting AR-mediated transcription. In HCT116 cells, PAK6 localizes in the cytoplasm, protecting cells from apoptosis through the phosphorylation of BAD 21. In DU145 and HT29 cells, PAK6 localizes in the cell-cell junctions, playing a role in cytoskeleton regulation by interacting with IQGAP1, in order to increase cell colony escape 22. However, it remains to be investigated whether PAK6 is located in the mitochondria, and how PAK6 affects mitochondrial function.

SIRT4 is a mitochondrial matrix protein, is a member of the sirtuin (SIRT) family, and serves as both ADP ribonucleotransferase and lysine deacetylase 23-27. Studies have reported that SIRT4 is extremely sensitive to DNA damage and changes in cell nutrients 28-30. When DNA damage occurs in cells, SIRT4 expression is increased, and it stops glutamine metabolism, blocks the cell cycle, and inhibits tumor formation 28. When SIRT4 is absent, the cell will maintain division even when DNA damage occurs, making the cell accumulate more DNA damage, and eventually lead to tumor occurrence. The low expression of SIRT4 in various tumor tissues has been reported, which is closely correlated to poor prognosis 28, 31-34. However, few is known about the roles of SIRT4 in prostate cancer.

Adenine nucleotide translocase-2 (ANT2) has the most abundant expression in the mitochondrial inner membrane, and is specifically expressed in proliferating tissues or tumor cells 35-38. Unlike ANT1 and ANT3, which export ATP from mitochondrial oxidation phosphorylation to the cytoplasm, ANT2 inputs the ATP produced by glycolysis to the mitochondria and ADP into the cytoplasm 35, 36. ANT2 maintains mitochondrial cross membrane potential (ΔψM), preventing mitochondrial membrane rupture, reducing reactive oxygen species (ROS) release, and inhibiting mitochondrial apoptosis, in order to promote tumor cell growth and resist chemotherapy 38, 39. It has been reported that the expression of ANT2 is associated with cell growth, and is a hallmark of cell proliferation 40, 41. Although most literatures have reported that the silencing of ANT2 is effective in suppressing multiple tumors 42, 43, there is little study on how ANT2 is involved in prostate cancer.

In the present study, it was found that PAK6 promotes SIRT4 ubiquitin-proteasomal degradation, and that SIRT4 relies on its deacetylation activity to promote ANT2 ubiquitination degradation. Therefore, PAK6 regulates the stability of ANT2 by regulating SIRT4 deacetylation activity. Meanwhile, PAK6 directly phosphorylates ANT2 at T107 to inhibit the apoptosis of prostate cancer cells.

Therefore, the phosphorylation and deacetylation modifications of ANT2 are mutually regulated, leading to tumor growth *in vivo*. Importantly, it was verified that PAK6 and ANT2 are highly expressed in cancerous tissues, and that SIRT4 is lowly expressed, Furthermore, PAK6 is positively correlated with ANT2 expression, but negatively correlated with SIRT4. These findings suggest that the PAK6-SIRT4-ANT2 complex plays a key role in apoptosis in prostate cancer.

## Materials and methods

### Cell culture and Transfections

The human embryonic kidney HEK-293 cell line was cultured in Dulbecco's modified Eagle's medium (DMEM) containing 10% fetal bovine serum (FBS). Prostate cell lines CWR22Rv1 and PC3 were cultured in RPMI 1640 medium containing 10% FBS. For the glutamine deficiency experiment, cells were maintained in Advanced RPMI 1640 medium, which had no glutamine (Gibco lot: 12633012).Lipofectamine 3000 (Invitrogen) was used for transfection. Cells were co-transfected with the listed constructs, according to the manufacturer's instructions. After 6 hours of transfection, the medium was changed to glutamine-free medium for 48 hours.

Then, the cells were collected for subsequent experiments.

### Plasmid constructs

pcDNA-EGFP-PAK6 wild-type (WT)/K436A (KA, kinase-dead) were gifts from Dr. G. M. Bockoh (The Scripps Research Institute). Flag/GST-tagged PAK6 and PAK1,4,5 were constructed by polymerase chain reaction (PCR) and subcloned into pcDNA3.1-Flag (Invitrogen) and pGEX-5X-1/2 (GE Healthcare) vectors, respectively. Flag-tagged SIRT4 was purchase from GeneChem (Shanghai, China). HisA-ANT2 WT was constructed by PCR and subcloned into pcDNA3.1/Myc-HisA (Invitrogen) vectors, respectively. The site-directed mutagenesis (ANT2 T107A mutant, ANT2 K105R mutant and ANT2 T107A/K105R mutant) was generated from ANT2 WT using the QuikChange kit (Stratagene), according to the manufacturer's instructions. The myc-ubiquitin constructs were previously generated in our laboratory.

### Lentiviral production and infection

PAK6-lentivirus (PAK6-Lv) and PAK6 shRNA-lentivirus (shPAK6:5'-AGTGATCTCCAGGTCTTTGTA-3'), and ANT2 WT-lentivirus (ANT2 WT-Lv), ANT2 T107A-lentivirus (ANT2 T107A-Lv), ANT2 T107A/K105Rlentivirus (ANT2 T107A/K105R-Lv) and ANT2 shRNA-lentivirus (shANT2:5'-GCCTACTTCGGTATCTATG-3') were produced by GeneChem (Shanghai, China). Stable cell lines were obtained. These cells were infected with lentiviral supernatants for 24 hours, and selected with 2 μg/ml of puromycin (Sigma, St. Louis, USA) for 48 hours, according to manufacturer's instructions. Then, the infected cells were passaged before use after identification by western blot.

### RNA isolation and qRT-PCR

Total RNA was isolated using Trizol reagent (Invitrogen, Carlsbad, USA). Total RNA (2 μg) was used for the synthesis of first-strand cDNA using M-MLV reverse transcriptase (Invitrogen, Beijing, China). Quantitative real-time PCR was performed using the SYBR green mix (Applied Biosystems). The reactions were performed using a 7500 Fast Real-Time PCR System (Applied Biosystems). The sequences of the RT-PCR primers were as follows (5′-3′): PAK6 (GACTCCATCCTGCTGACCCTC and CACCTCAGTGGCATACAAAGACC), SIRT4 (GACAAGGTTGATTTTGTGCAC and TCAAAGGCAGCAACTCTCCAC), and GAPDH (GCCAAAAGGGTCATCATCTC and CTAAGCAGTTGGTGGTGCAG).

### Immunoprecipitation, immunoblotting and GST pull-down assays

For immunoprecipitation, cells were lysed with 500 μl of lysis buffer containing protease and phosphatase inhibitors (Sigma, St Louis, USA) for 30 minutes at 4°C after washing with cold PBS twice and centrifugation at 12,000 *g* for 20 minutes at 4°C. The total protein in whole-cell extracts was measured using the Bradford method, equal amounts of lysate (2 mg) were used for the immunoprecipitation with the indicated antibodies and protein A-Sepharose (GE Healthcare, USA), and these were incubated overnight at 4°C. Then, the washed precipitated proteins were analyzed by western blot. The immunoprecipitation, western blot and GST pull-down assays used in the present study were previously described in detail 44.

### Antibodies and reagents

Antibodies against the following proteins were used in the experiments: PAK6 (Cell Signaling; Santa Cruz Biotechnology, Abcam), ANT2 (Cell Signaling, R&D Systems, Minneapolis, USA), SIRT4 (Santa Cruz Biotechnology, Abcam), COX IV (Cell Signaling), cleaved-caspase 3 and 9 and PARP (Cell Signaling), acetylated-lysine antibody (Cell Signaling), c-Myc-tag and Flag-tag M2 (Sigma-Aldrich), His-tag and GFP-tag (GenScript Corporation), Actin (KangChen Bio-tech), and MG-132 (Sigma-Aldrich).

### Immunofuorescence

Cells were fixed in 4% paraformaldehyde for 20 minutes at room temperature and sealed with normal goat serum for 30 minutes. After washing for three times in PBST (PBS containing 1‰ Triton X-100), these cells were incubated overnight with the primary antibody at 4°C, and incubated with Alexa Fluor 488 (green) and 546 (red) dye conjugated with Moleculara Probes. The DNA dye DAPI (molecular probe, blue) was used. The confocal scanning analysis was performed with a Ultraview Vox Spinning disc confocal microscope (USA, Perkin Elmer) , in order to minimize the possibility of leakage of fluorescence emission.

### Mitochondrial protein extraction

In order to purify the mitochondrial protein, a Cell Mitochondria Isolation Kit (C3601, Beyotime) was used, according to the manufacturer's instructions. Then, the cells were collected, washed with precooled PBS, added with the appropriate amount of mitochondrial separation reagent, and homogenized in a glass homogenizer for 50 times. Afterwards, the supernatant was centrifuged at 1,000 × g at 4°C to obtain the required mitochondrial protein. Finally, 30 μl of concentrated protein was used for the western blot.

### Ser/Thr phosphoprotein purification assay

In order to purify the Ser/Thr phosphoprotein, a PhosphoProtein Purification Kit (Qiagen no. 37101) was used, according to manufacturer's instructions. A certain volume of lysates that contained 2.5 mg of total protein was taken, and the protein concentration was adjusted to 0.1 mg/ml. Finally, 30 μl of concentrated protein was used for the western blot 45.

### Immunoelectron microscopy

Cells were fixed in 1% paraformaldehyde overnight at 4°C, and 1% wt/vol gelatine in PB collected cells were transferred to EP tubes, resuspended in 12% gelatin after centrifugation, allowed to stand at 37°C for 5 minutes, and centrifuged again at 4°C for 20 minutes. Then, the cut, sliced and reserved cells were incubated with the primary antibody overnight at 4°C, colloidal-gold-labeled with protein A, and uranium-dyed. After drying, the dried tablets are observed by transmission electron microscopy 46, 47.

### Ubiquitination assay

CWR22RV1 cells and PC3 cells were transfected with or without the myc-ubiquitin constructs encoded in the indicated plasmids, and treated with 5 uM of MG132 for 12 hours. At 48 hours after transfection, these cells were harvested and sonicated in ubiquitination-lysis buffer with 250 ng/ml of ubiquitin-aldehyde. Then, western blot analysis was performed to evaluate the protein degradation.

### Cell cycle assay

After allowing these cells to adhere for 12 hours, these cells were trypsinized. Then, these cells were gently collected using PBS. After centrifugation, these cells were resuspended in 75% alcohol, fixed at 4°C for 24 hours, washed with PBS, and stained with propidium iodide (PI) at 37°C for 30 minutes. Flow cytometry was performed to detect the cell cycle.

### Tumor xenograft analysis

Next, 5-6 weeks old male NOD/SCID nude mice (average body weight: 20-25 g) were injected with 100 ul of Matrigel (BD Biosciences) plus 1×106 PC3 cells in the left axilla, and the underground nodules were observed after 4 weeks of injection. The tumor size was measured using a caliper. Then, mice were sacrificed after 8 weeks, photographed, the final weight of the tumor was weighed, and the standard subcutaneous nodules were 0.5 cm.

### Immunohistochemistry and patient tissues

Prostate cancer tissue chips were purchased from Shanghai SuperChip Biotech Co. Ltd. These chips were stained with the Dako Liquid 3,3′-diaminobenzidine tetrahydrochloride (DAB) + Substrate Chromogen system, and counterstained withhematoxylin. The immunohistochemical staining of paraffin-embedded tumor tissues was performed using the appropriate primary and secondary antibodies, and the ABC Elite immunoperoxidase kit, according to manufacturer's instructions. The H-score (histological score) was used to evaluate the staining results. The prostate cancer tissue specimens collected between 2018 and 2019 were obtained from the Department of Urology of the First Affiliated Hospital of China Medical University. All tissues were collected at the time of surgical resection and immediately stored in liquid nitrogen until protein extraction for western blot. The patient clinical characteristics were collected and recorded. The work described in the present study was carried out in accordance to The Code of Ethics of the World Medical Association (Declaration of Helsinki).

### Statistical analysis

The GraphPad Prism software was used for the statistical analysis. Student's *t-*test and one-way analysis of variance were performed to determine the statistical significance among values in the *in vitro* experiments. The data derived from the immunostaining analysis of human prostate tissue specimens were analyzed using paired *t-*test. *P*<0.05 was considered statistically significant, and *P*<0.01, *P*<0.001 and *P*<0.0001 were considered highly significant.

## Results

### PAK6 is associated with the mitochondrial inner membrane

**Figure 1:**
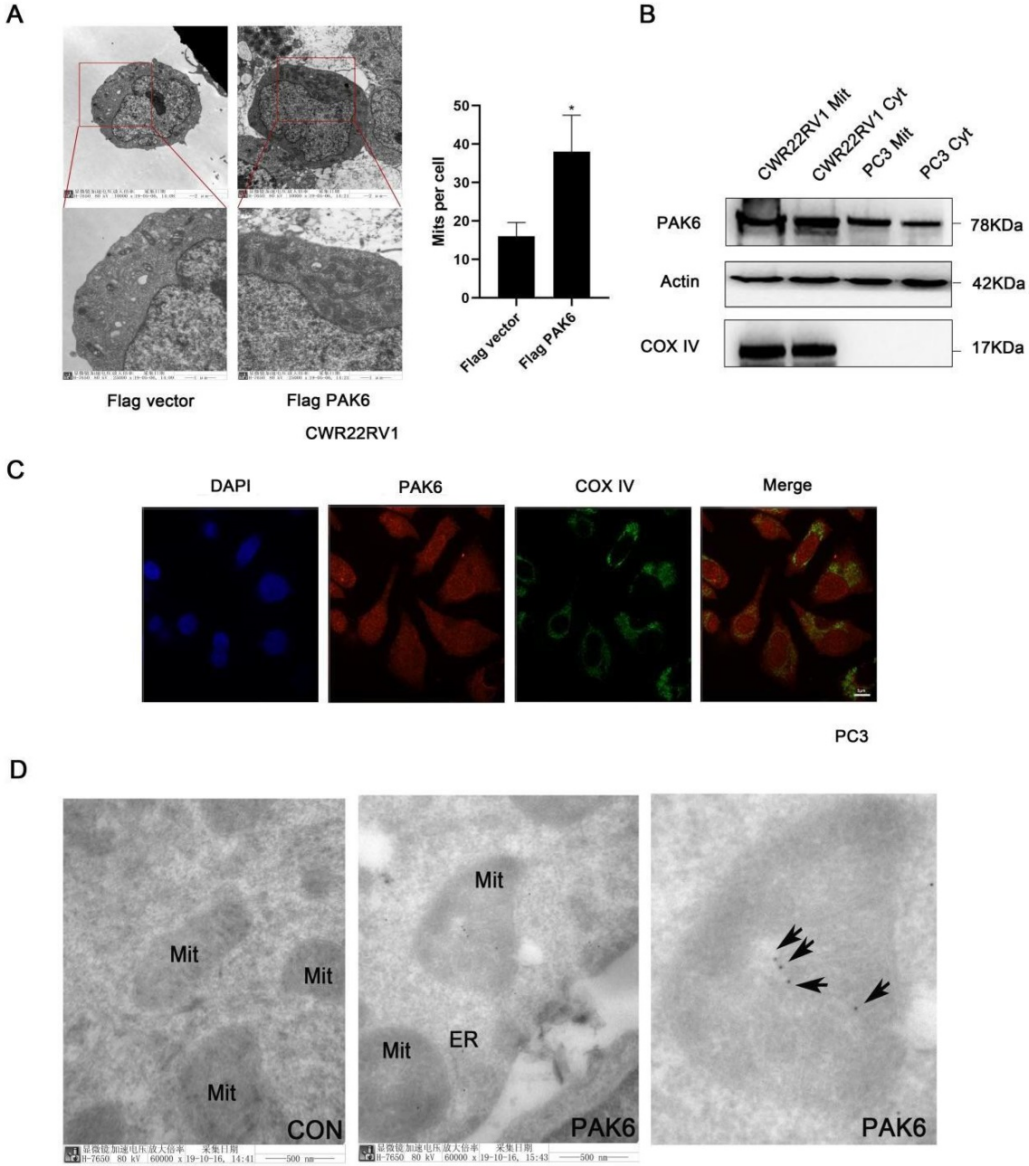
PAK6 is associated with the mitochondrial inner membrane. (A) Cells that stably expressed the Flag vector and Flag-PAK6 were collected and stained. The representative images obtained by the electron microscope are shown. The data are presented as a histogram of the mean ± standard error of the mean (SEM) of five independent cells (Student's *t*-test). (B) PAK6 is expressed in the mitochondria. The extracted mitochondrial proteins, and the western blot analysis of mitochondria and other cellular components are shown. (C) The co-localization of endogenous PAK6 (red), COX IV (green), and the nuclei (DAPI blue). The merged images with the nucleus are shown, as indicated. Original magnification: ×40. (D) Immunogold labeling of a cryosection of prostate cancer cell CWR22RV1 for PAK6 (10 nm gold). Original magnification: ×60,000.

In order to investigate the role of PAK6 in prostate cancer cells, the effect of PAK6 on the subcellular structure of prostate cancer cell CWR22RV1 was initially observed using transmission electron microscopy. The results revealed that the number of mitochondria significantly increased (Figure 1A, *P*=0.0245) when PAK6 was ectopically expressed in CWR22RV1 cells. In order to further investigate the correlation between PAK6 and the mitochondria of prostate cancer cells, CWR22RV1 and PC3 cells were used as a model system in the subsequent studies. By extracting mitochondrial proteins from CWR22RV1 and PC3, the results revealed that PAK6 protein was expressed in the mitochondria (Figure 1B). The confocal laser scanning microscopy revealed that PAK6 co-localized with COX IV protein which is a mitochondrial inner membrane marker (Figure 1C). In order to further explore the specific localization of PAK6 in the mitochondria, PAK6 was indirectly labeled with 10 nm of colloidal gold by immunoelectron microscopy. The results revealed that PAK6 was mainly located in the mitochondrial inner membrane (Figure 1D). The above data indicates that PAK6 increases the number of cellular mitochondria and localizes to the mitochondrial inner membrane.

### PAK6 promotes the ubiquitination degradation of SIRT4

**Figure 2:**
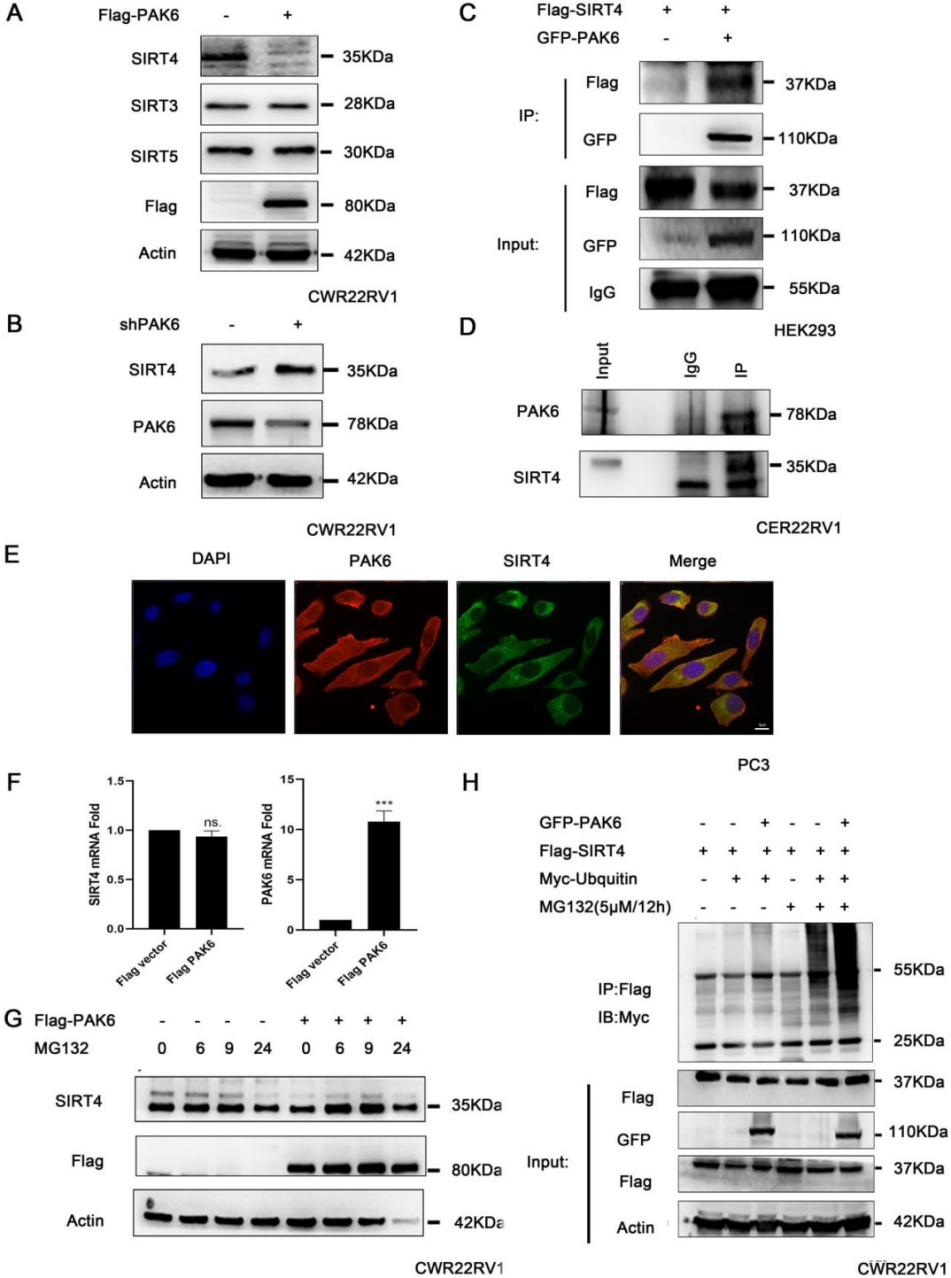
PAK6 promotes the ubiquitination degradation of SIRT4. (A-B) CWR22RV1 cells were transfected with Flag-tagged PAK6 or shPAK6. SIRT3, SIRT4 and SIRT5 protein expression levels were determined by western blot. (C) HEK293 cells that expressed Flag-tagged SIRT4 were co-transfected with or without GFP-PAK6, and endogenous PAK6 and SIRT4 were evaluated in CWR22RV1 cells.(D) The PAK6-SIRT4 interaction was identified through immunoprecipitation and western blot. (E) The co-localization of endogenous PAK6 (red), SIRT4 (green), and the nuclei (DAPI blue). Merged images with the nucleus are shown, as indicated. Original magnification: ×40. (F) CWR22RV1 cells stably overexpressed PAK6 were harvested. The total RNA was analyzed by RT-qPCR. The levels of all mRNAs were normalized to that of β-actin mRNA. The statistical significance of the differences between experimental groups was assessed by one-way ANOVA. The error bars represent the mean ± standard error of the mean (SEM). *P<0.05, **P<0.01 and ***P<0.001. (G-H) Cells that overexpressed PAK6 were incubated with medium containing 5 μM of MG132 for the indicated time points. SIRT4 expression levels were determined by western blot and immunoprecipitation, with the indicated antibodies. MG132 was stimulated at 24 hours due to drug toxicity, and cell death was strictly heavy. Hence, all protein levels were low, and there was no reference value.

In order to gain mechanistic insight into the function of mitochondrial PAK6 in prostate cancer cells, based on the previous study of the investigators, which revealed that PAK5-mediated GATA1 phosphorylation is linked to histone deacetylation in E-cadherin transcription regulation, it was speculated that class II PAKs are closely correlated to the deacetylase family 45. Therefore, after the overexpression of PAK6, the deacetylase family of SIRTs in the mitochondria, which included SIRT3, SIRT4 and SIRT5, was examined. The western blot analysis revealed that PAK6 specifically downregulated the expression of SIRT4, but did not regulate SIRT3 and SIRT5 in cells with a stable overexpression of Flag-PAK6 (Figure 2A). In the meantime, the PAK6 knockdown led to opposing effects (Figure 2B). In order to study the specific correlation between PAK6 and SIRT4, a coimmunoprecipitation (coIP) assay was performed to validate the PAK6-SIRT4 interaction. HEK293 cells were transiently transfected with GFP-PAK6 and Flag-SIRT4, and immunoprecipitation with an anti-GFP antibody was performed. As shown in Figure 2C, PAK6 coimmunoprecipitated with SIRT4. Similarly, the reciprocal immunoprecipitation with the endogenous SIRT4 antibody brought down PAK6 (Figure 2D). Furthermore, the immunofluorescence staining of PAK6 and SIRT4 revealed that there was a remarkable co-localization in both of these in the cytoplasm in PC3 (Figure 2E).Nonetheless, this regulation did not occur on the transcription level (Figure 2F). This result suggests that PAK6 may regulate the expression of SIRT4 through post-translational modification. In order to determine its specific regulation mechanism, PAK6-overexpressing cells were exposed to MG132, which is a well-established inhibitor of the ubiquitin-proteasome pathway, for the indicated time points. Consistent with the assumptions of the investigators, the SIRT4 expression obviously accumulated. Furthermore, MG132 was stimulated at 24 hours due to drug toxicity, and cell death was strictly heavy. Hence, all proteins were at the low level, without a reference value (Figure 2G). Moreover, the PAK6 overexpression exhibited a marked ubiquitin-linked SIRT4 degradation in the presence of MG132 (Figure 2H). These data suggest that the overexpression of PAK6 promotes the ubiquitination degradation of SIRT4.

### SIRT4 promotes the ubiquitination degradation of ANT2 depending on its deacetylation activity

**Figure 3:**
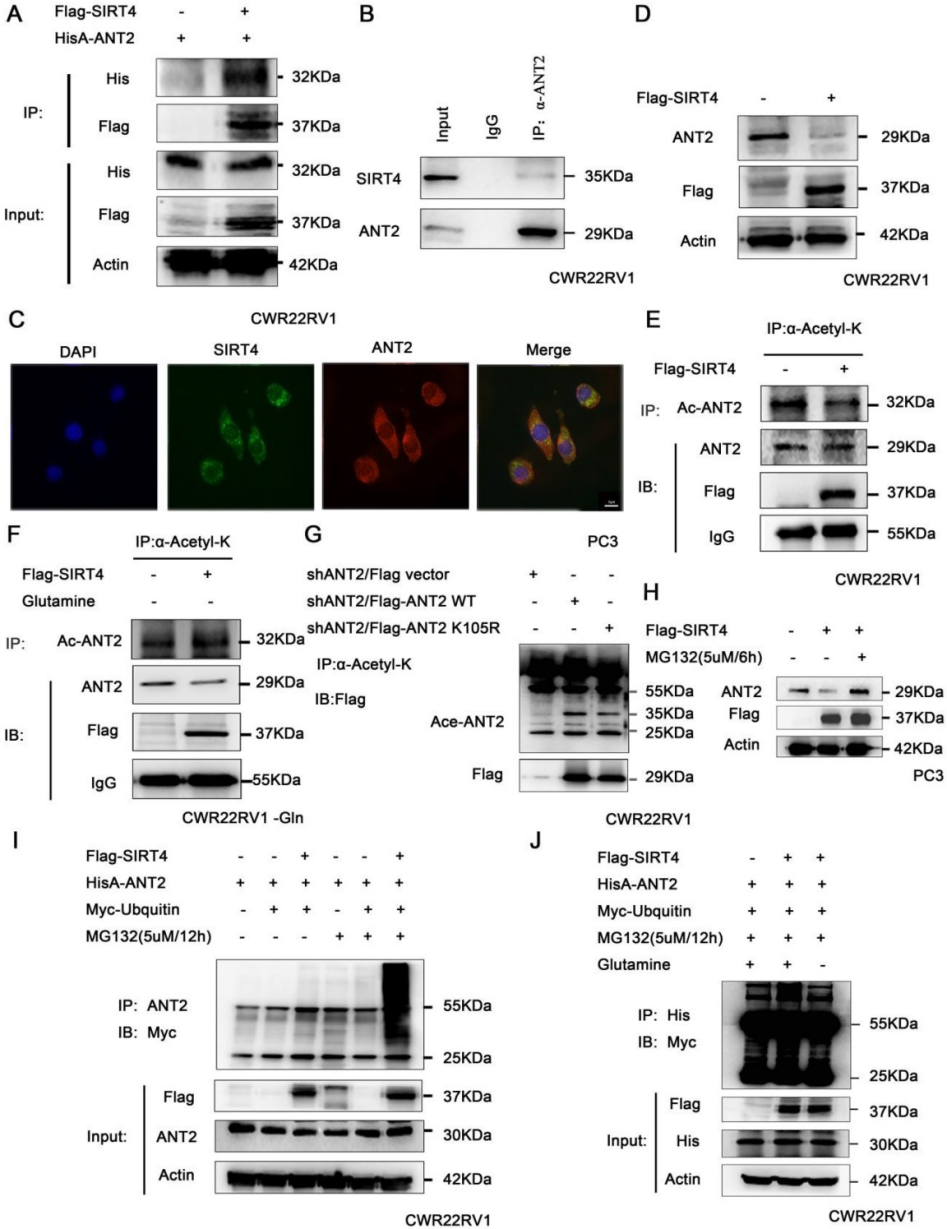
SIRT4 promotes the ubiquitination degradation of ANT2 depending on its deacetylation activity. (A) HEK293 cells expressing HisA-ANT2 were co-transfected with or without Flag-tagged SIRT4, and (B) the endogenous SIRT4 and ANT2 were evaluated in CWR22RV1 cells. The SIRT4-ANT2 interaction was identified through immunoprecipitation and western blot. (C) The co-localization of endogenous SIRT4 (red), ANT2 (green), and the nuclei (DAPI blue). The merged images with the nucleus are shown, as indicated. Original magnification: ×40. (D) CWR22RV1 cells were transfected with Flag-tagged SIRT4. The protein expression levels were determined by western blot. (E-F) CWR22RV1 cells were transfected with Flag-tagged SIRT4, with or without glutamine, the broad-spectrum acetylated antibody was enriched for acetylated protein, and the Ac-ANT2 protein expression levels were determined by immunoprecipitation and western blot. (G) The indicated ANT2 residues were mutated, as indicated (K105R), and the resulting proteins analyzed via immunoprecipitation and western blot. (H-I) Cells that overexpressed SIRT4 were incubated with a medium containing 5 μM of MG132 for 12 hours. The ANT2 expression levels were determined by western blotting and immunoprecipitation with the indicated antibodies. (J) CWR22RV1 cells that were transfected with the indicated constructs were exposed to 5 μM of MG132 for 12 hours in culture medium, with or without glutamine. Western blot was performed with the indicated antibodies.

It has been reported that deacetylase SIRT4 may regulate the level of ANT2 protein by deacetylation, and maintain the mitochondrial energy balance by coupling with ANT2 23, 24. Hence, the association of SIRT4 and ANT2 was verified by immunoprecipitation in prostate cancer cells (Figure 3A-B). At the same time, the immunofluorescence staining of SIRT4 and ANT2 revealed that there was a remarkable co-localization in both of these in the cytoplasm in PC3 (Figure 3C). Moreover, the overexpression of SIRT4 downregulated the protein expression of ANT2 (Figure 3D). Since SIRT4 is one of the family of deacetylases, we sought to determine its role in ANT2 acetylation activity. The immunoprecipitation results revealed that SIRT4 overexpression downregulated the ANT2 acetylation (Figure 3E). Since SIRT4 is a potent regulator of glutamine metabolism, and prostate cancer is a tumor described as "glutamine addiction"^48^, ^49^. Therefore, it was speculated that glutamine is likely to be an important factor in the regulation of ANT2 by SIRT4. To this end, prostate cancer cells without glutamine were cultured for 48 hours, and it was found that the regulation of acetylation activity of ANT2 by SIRT4 was dependent of the presence of glutamine in culture medium (Figure 3F). Furthermore, based on the bioinformatics and the present experimental results, it was found that K105 is one of the acetylation sites of ANT2 (Figure 3G). Moreover, the addition of MG132 effectively inhibited the degradation of SIRT4 to ANT2 (Figure 3H).Consistent with these present assumptions, SIRT4-overexpressing cells exhibited a marked ubiquitin-linked ANT2 degradation in the presence of MG132 for the indicated time points (Figure 3I). In order to determine the correlation between the two modifications of ANT2 protein, after the overexpression of SIRT4, the accumulation of ubiquitin-linked ANT2 was significantly attenuated in the absence of glutamine (Figure 3J). The results suggest that the ubiquitination degradation of ANT2 by SIRT4 depends on its deacetylation activity.

### PAK6 phosphorylates ANT2 at T107 and affects the acetylation of ANT2

**Figure 4:**
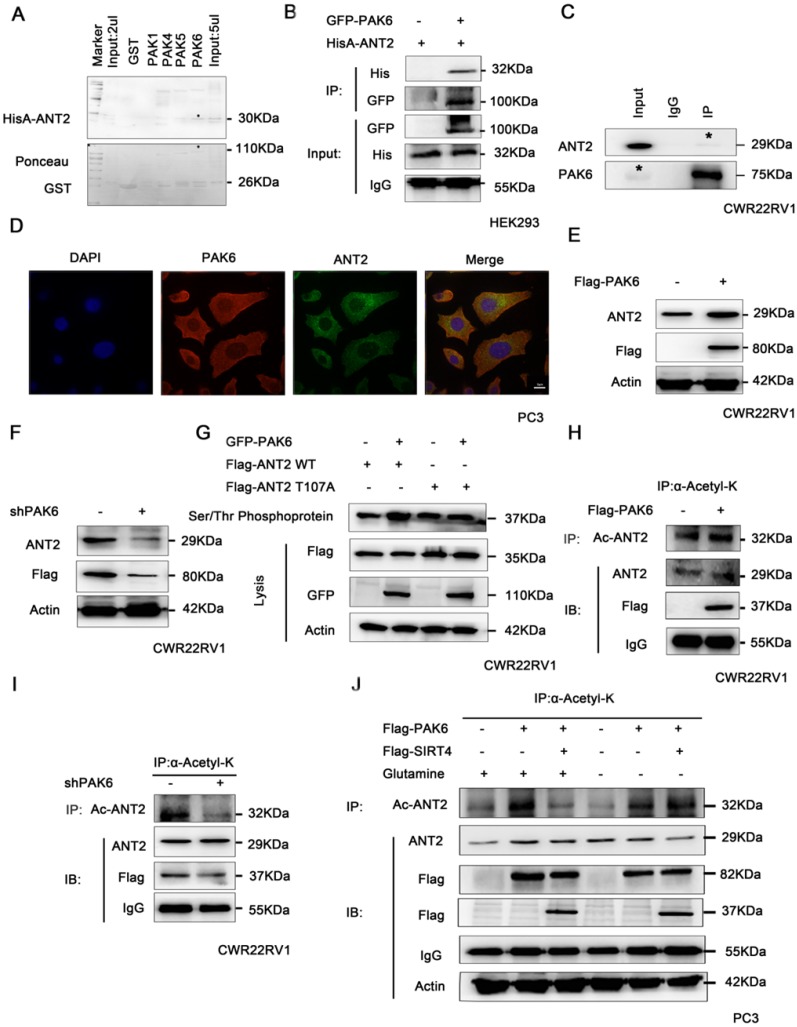
PAK6 phosphorylates ANT2 at T107 and affects the acetylation of ANT2. (A) Recombinant human ANT2 was incubated with bacterially expressedGST-PAK1/4/5/6, and western blot was performed to evaluate the interaction. (B) HEK293 cells expressing His-ANT2 were co-transfected with or without GFP-taggedPAK6, and (C) endogenous PAK6 and ANT2 were evaluated in CWR22RV1 cells. The PAK6-ANT2 interaction was identified by immunoprecipitation and western blot. (D) The co-localization of endogenous PAK6 (red), ANT2 (green), and the nuclei (DAPI blue). The merged images with the nucleus are shown, as indicated. Original magnification: ×40. (E-F) CWR22RV1 cells were transfected with 1 ug of Flag-PAK6 plasmid or shANT2, and the western blot analysis was performed. (G) CWR22RV1 cells were transfected with ANT2 WT/S107A and PAK6 WT, and were used for Ser/Thr phosphoprotein purification. Then, the concentrated protein was used for western blot. On the top lane, the phosphorylated ANT2 from cell lysates was used for immunoblotting with the anti-Flag antibody. Total cell lysates were used for immunoblotting with anti-Flag, GFP and actin antibodies. (H-I) CWR22RV1 cells were transfected with Flag-tagged PAK6 or knockdown PAK6, the broad-spectrum acetylated antibody was enriched for the acetylated protein, and the Ac-ANT2 protein expression levels were determined by immunoprecipitation and western blot. (J) CWR22RV1 cells were transfected with Flag-tagged PAK6 with or without Flag-tagged SIRT4, cells were cultured with or without glutamine, the broad-spectrum acetylated antibody was enriched for the acetylated protein, and the Ac-ANT2 protein expression levels were determined by immunoprecipitation and western blot.

It has been proven that PAK6 can degrade SIRT4, thereby stabilizing the protein expression level of ANT2. Hence, it remains to be determined whether PAK6 directly interacts with ANT2. In order to gain mechanistic insight into the association between PAK6 and ANT2, a GST pull-down assay as performed, it was revealed that ANT2 directly interacted with PAK4, PAK5 and PAK6 *in vitro*, and the strongest association was with PAK6 (Figure 4A). Furthermore, a co-immunoprecipitation assay was performed to validate the PAK6-ANT2 interaction. As shown in Figure 4B, PAK6 co-immunoprecipitated with ANT2. Similarly, the reciprocal immunoprecipitation with the endogenous PAK6 antibody brought down ANT2 (Figure 4C). Meanwhile, the immunofluorescence staining of PAK6 and ANT2 revealed that there was a remarkable co-localization in both of these in the cytoplasm in PC3 cells (Figure 4D). Moreover, PAK6 overexpression upregulated the protein expression of ANT2 (Figure 4E), while PAK6 knockdown led to opposing effects (Figure 4F). In view of the serine/threonine kinase activity of PAK6, a Serine/Threonine phosphoprotein purification kit was used to further test the PAK6-mediated ANT2 phosphorylation in cells (Figure 4G). The total serine/threonine phosphorylated protein from cell lysates were analyzed by western blot. The results revealed that the phosphorylated wild-type ANT2, but not the ANT2 T107A mutant, increased after the overexpression of PAK6. In addition, the overexpression of PAK6 indirectly increased the acetylation of ANT2 (Figure 4H), while the silencing of PAK6 decreased its acetylation (Figure 4I). The final rescue experiments confirmed that PAK6 regulates the acetylation of ANT2 by modulating SIRT4, and that PAK6 regulates the acetylation of ANT2, independent of glutamine (Figure 4J).

### The interaction between ANT2 phosphorylation and acetylation

**Figure 5:**
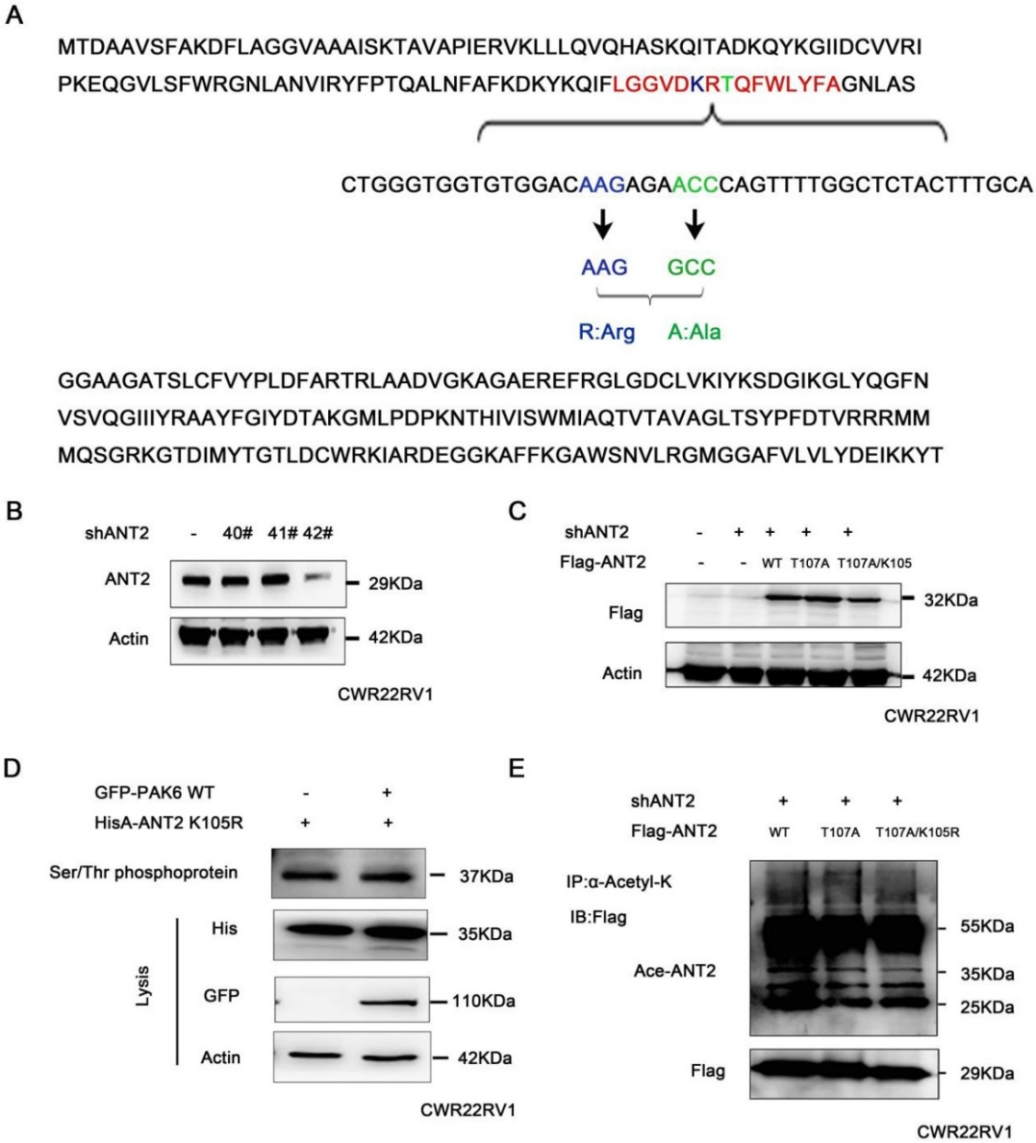
The interaction between the acetylation and phosphorylation of ANT2. (A) The amino acid and nucleotide sequence of ANT2 was analyzed. The acetylation site K105 was mutated to R;Arg (blue), and the phosphorylation site T107 was mutated to A;Ala (green). (B) CWR22Rv1 cells were infected with lentiviruses harboring shRNA control and shANT2 40#, 41# and 42#. (C) The 42# clone of ANT2 knockdown cells was selected, as shown in (B), and different types of stableANT2-expressing cell lines were constructed using the lentivirus, including wild-type(WT), dead-type phosphorylation (T107A), and mutant (T107A/K105R). (D) CWR22RV1 cells transfected with ANT2 K105R and PAK6 WT were used for the Ser/Thr phosphoprotein purification. Then, the concentrated protein was used for western blot. On the top lane, the phosphorylated ANT2 from cell lysates was used for immunoblotting with the anti-His antibody. The total cell lysates were used for immunoblotting with anti-His, GFP and actin antibodies. (E) CWR22RV1 cells were transfected with ANT2 WT, T107A and T107A/K105R, the broad-spectrum acetylated antibody was enriched for the acetylated protein, and the Ac-ANT2 protein expression levels were determined by immunoprecipitation and western blot.

These above studies revealed that PAK6 phosphorylated the ANT2 sites at T107 using the bioinformatics software combined with the prediction of phosphorylation motifs of Class II PAK. The predicted possible acetylation sites of ANT2 include K105 and K163. Since the phosphorylation and acetylation modification of proteins can interact 50, and the predicted ANT2 acetylation site K105 is adjacent to the phosphorylation site T107 (LGVDKRTQFWLYFA, Figure 5A), it was speculated that there may be a mutual regulation. Given that ANT2 was highly expressed in prostate cancer cells, ANT2 was silenced in prostate cancer cell PC3 with the 42# shANT2 clone, and these were selected and subjected to the subsequent experiments (Figure 5B). Then, single-site mutations of the predicted sites in ANT2 were created. As shown in Figure 5C, the mutations of Thr107 to alanine or K105 to argnine significantly impaired the ANT2 phosphorylation through PAK6 or ANT2 deacetylation by SIRT4. A stable ANT2-expressing cell line, including wild-type, dead type of phosphorylation (T107A) and mutants (T107A/K105R), was established (Figure 5C). Then, a Serine/Threonine phosphoprotein purification kit was used to further determine whether K105-acetylation regulated the phosphorylation of ANT2. Next, CWR22RV1 cells were transiently transfected with GFP-PAK6 andHisA-ANT2 K105R plasmids, and the ANT2 phosphorylation was abolished, regardless of the overexpression of PAK6 (Figure 5D). Furthermore, acetylated antibody was used to precipitate the acetylated protein in cells, and result revealed that the ANT2T107A mutation also blocked the acetylation of ANT2 (Figure 5E). In summary, the interplay existed between the T107 and K105 of ANT2, which was proven as a dual substrate of PAK6 and SIRT4.

### The PAK6-SIRT4-ANT2 complex affects the apoptosis of prostate cancer cells

**Figure 6:**
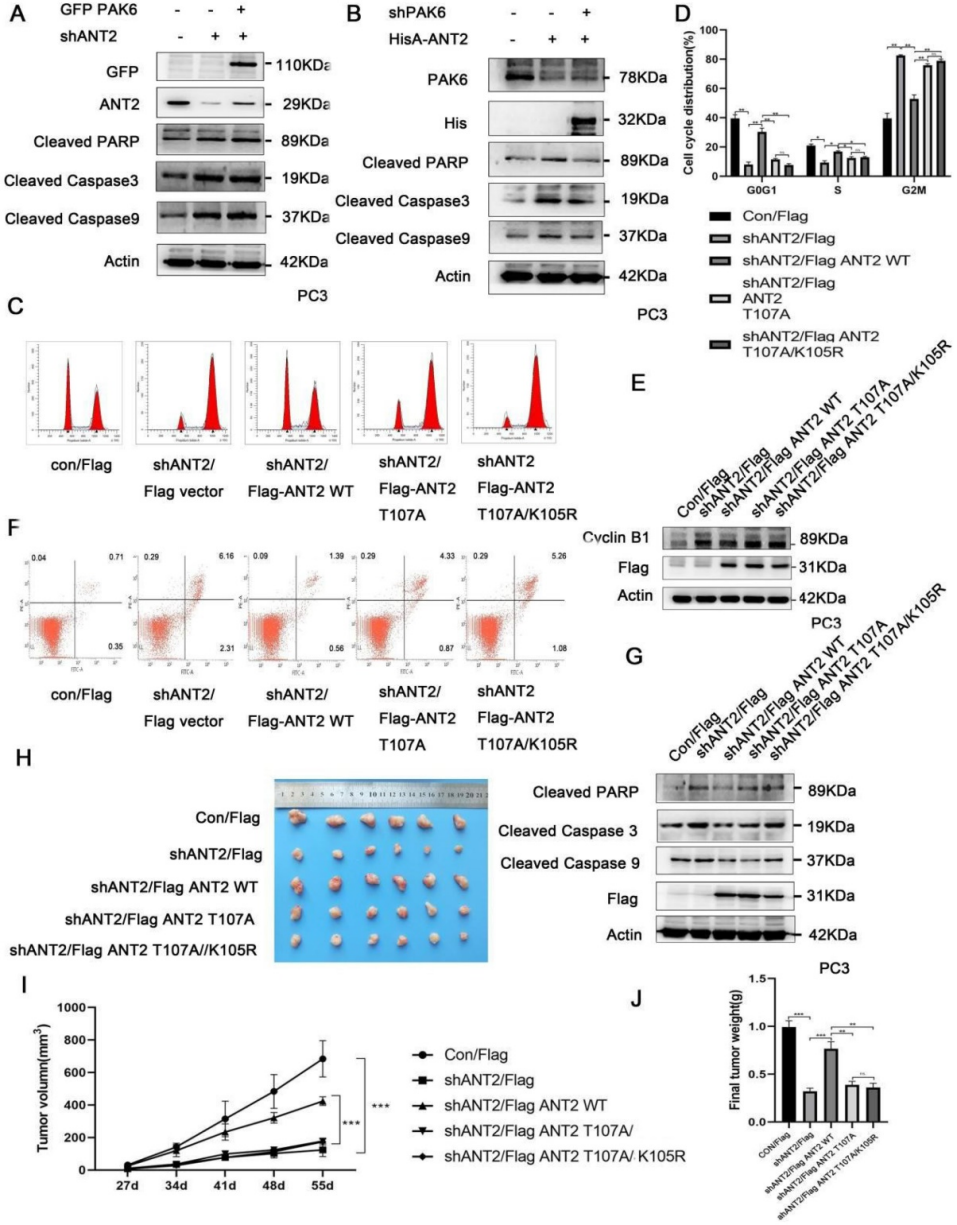
The PAK6-SIRT4-ANT2 complex inhibits the apoptosis of prostate cancer cells. (A) The shRNA control/PAK6 in CWR22Rv1 cells was infected with or without His-ANT2, (B) the shRNA control/ANT2 in CWR22Rv1 cells was infected with or without GFP-PAK6, and equal amounts of protein lysates were harvested for western blot with antibodies, as indicated. (C-D) The flow cytometry detected the relevant changes in the cell cycle of different mutant CWR22RV1 cells, as indicated. The data are presented as a histogram of the mean ± standard error of the mean (SEM) of three independent experiments (Student's *t*-test). (E) The western blot analysis of the G2/M phase protein marker CyclinB1. (F) The CWR22RV1 cells transfected with different mutant ANT2 is shown after double staining with annexin V-FITC and propidium iodide. The dotted plot shows the annexin V-FITC in the X-axis and propidium iodide in the Y-axis (%). (G) CWR22RV1 cells were transfected with different mutant ANT2, and equal amounts of protein lysates were harvested for western blot with antibodies, as indicated. (H-J) Mice were subcutaneously injected with 1×10^6^of CWR22RV1cells with lentiviruses harboring shRNA, with or without the ANT2 mutant, and were grouped. The tumor volume was monitored over time, and the tumor was excised and weighed after 55 days.

In order to determine the association of the PAK6-SIRT4-ANT2 complex with apoptosis, the apoptosis indexes of cancer cells were evaluated. The apoptosis-related protein cleaved caspase3/9 and PARP were tested, since PAK6, SIRT4, or ANT2 has been separately reported to be correlated to apoptosis 11, 51, 52. In the experiment, PAK6 or ANT2 was silenced, and activated caspase 3 and 9, and PARP significantly increased, suggesting that the apoptosis increased in prostate cancer cells. Based on the depletion of PAK6, the level of apoptosis in prostate cancer cells was recovered following the overexpression of ANT2 (Figure 6A). However, when ANT2 was knocked down, the level of apoptosis did not recover after PAK6 overexpression (Figure 6B), suggesting that PAK6 affects the apoptosis of prostate cancer cells through ANT2. As shown in Figures 6C-6E, the cell cycle experiments confirmed that the silencing of ANT2 reduced the cell cycle in the S phase, while most of the cells were arrested in the G2M phase. The addition of the K105 mutant has a slight effect, when compared with the single phosphorylation the T107 mutant, because ANT2 K105R has an effect on ANT2 phosphorylation. The G2/M phase marker protein CyclinB1 kept up with this change, which suggest that the phosphorylation of ANT2 plays a major role (Figure 6C-E). The flow cytometric analysis of PC3 cells after double staining with annexin V-FITC and PI revealed that the knockdown ANT2 increased cell apoptosis, and that the T107A and T107A/K105R mutants could not restore its level of apoptosis (Figure 6F). Furthermore, the simultaneous detection of activated caspase 3 and 9, and PARP was consistent with the cycle results (Figure 6G), suggesting that ANT2 phophorylation affects prostate cancer cell apoptosis. In addition, this conclusion was also verified *in vivo* using tumor xenografts (Figure 6H-J). These data indicate that ANT2 phosphorylation inhibits prostate cancer apoptosis.

### The association of the PAK6-SIRT4-ANT2 complex in prostate cancer

**Figure 7:**
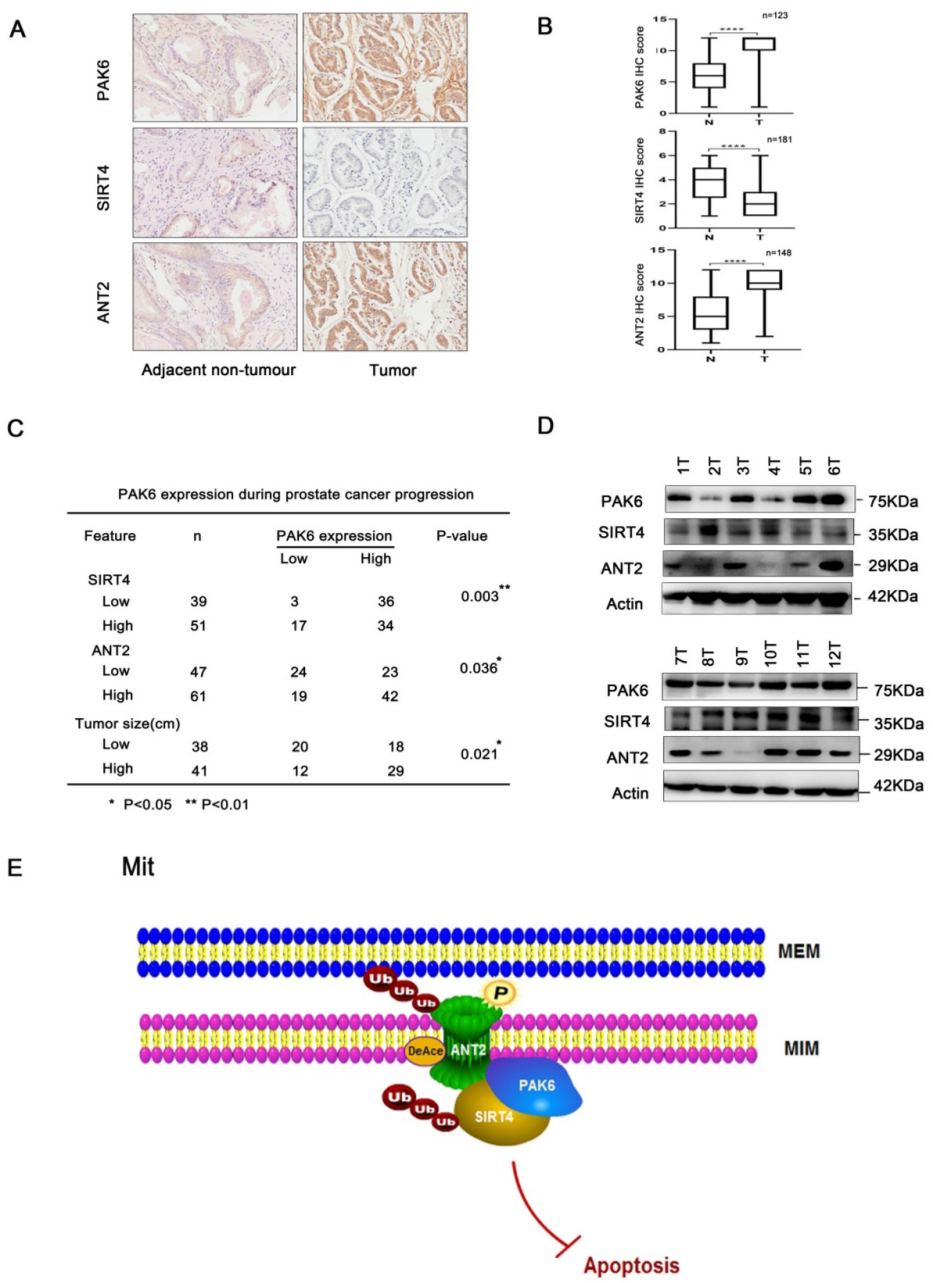
The association of the PAK6-SIRT4-ANT2 complex in prostate cancer. (A) Representative images of the immunohistochemical staining showing the PAK6, SIRT4 and ANT2 protein expression. Original magnification: ×200. (B-C) The prostate tumor specimens with paired adjacent non-neoplastic tissues were collected and subjected to immunofluorescence analysis. The immunohistochemistry (IHC) staining was scored, and Pearson's correlation test was performed. A total score (ranging from 0 to 12) was obtained by multiplying the staining intensity and fraction scores. **** means a *P-*value of ≤0.001 was considered statistically significant according to the unpaired *t-*test. (D) The lysates of the tumor tissues (T) were analyzed by western blot. A total of 12 representative specimens are shown. (E) The proposed model shows the role of the PAK6-SIRT4-ANT2 complex and its mechanism in prostate cancer. During the development of prostate cancer, the overexpression of PAK6 in the mitochondria promotes the ubiquitination of SIRT4, which in turn affects the deacetylation and ubiquitination of ANT2 through SIRT4. At the same time, PAK6 directly phosphorylates ANT2, and stabilizes and activates its function, which in turn affects the apoptosis of prostate cancer.

Next, further support was searched for the present discovery in human prostate cancer. The correlation of the PAK6-SIRT4-ANT2 complex in prostate cancer was investigated, and the expression levels of PAK6, SIRT4 and ANT2 were evaluated in prostate cancer. Dramatically, it was found that prostate cancer tissues had apparently higher PAK6 and ANT2 expression, but had dramatically lower SIRT4 expression, when compared to paired adjacent non-neoplastic tissues. In addition, the histopathologic analyses of these specimens revealed that a higher PAK6/ANT2 expression and a lower SIRT4 expression were significantly associated with the tumor size (Figure 7A-C). At the same time, in support of these present discoveries, it was also found that PAK6 and ANT2 are highly expressed, and that SIRT4 is lowly expressed in 9/12 frozen clinical prostate cancer tissues (Figure 7D). This findings verifies our previous cellular results *in vivo*. Taken together, these data are consistent with the functions of the PAK6-SIRT4-ANT2 complex, thereby providing strong support for these conclusions.

## Discussion

To the best of our knowledge, the present study is the first to reveal that PAK6 co-localized with the mitochondrial inner membrane in prostate cancer cells. Furthermore, it was found that multiplex post-translational modifications between proteins in the PAK6-SIRT4-ANT2 complex, including the promotion of ubiquitination degradation by de-acetylation, and the interplay between acetylation atK105 and phosphorylation at T107, leads to various modifications, which work together on the target protein ANT2. These in turn eventually regulates the apoptosis of prostate cancer (Figure 7E). Importantly, it was also found that PAK6 and ANT2 were highly expressed in cancerous tissue, SIRT4 was lowly expressed in cancerous tissues, and PAK6 was positively correlated with ANT2 expression, but negatively correlated with SIRT4. Taken together, these findings can help to better understand the molecular mechanism underlying PAK6 in mitochondria in inhibiting the apoptosis of malignant prostate cancer.

PAK6 is a member of the class II PAK family, and is involved in the progression of various malignancies 9, 13, 14, 17, 20. A study revealed that PAK6 is highly expressed in a variety of malignant tumors, including prostate cancer, colon cancer, ovarian cancer and lung cancer 53. The expression of PAK6 in prostate cancer tissues is significantly higher than that of normal prostate tissues, especially after desperation. Hence, PAK6 may play an important role in the movement of tumor cells, and may play an important role in stress response 17. Therefore, the study of the regulation mechanism of PAK6 has implications for the treatment of prostate cancer. Studies on the upstream regulators of PAKs have progressed, as follows. As it isknown, unlike the Group I PAKs that are mainly activated by small Rho-GTPases, including Cdc42 and Rac, it has been considered that Rho GTPases regulate class II PAK kinases by controlling the subcellular location, rather than directly stimulating the kinase activity 54. Even though PAK6 bounded strongly to GTP-Cdc42 and weakly to GTP-Rac, in contrast to most PAKs, the PAK6 kinase activity was not stimulated by Cdc42 or Rac, but this could be recruited by RhoD to the plasma membrane, in order to antagonize RhoC signaling during cell contraction and blebbing 16, 55. Furthermore, this could be stimulated by AR binding in androgen-dependent prostate cancer, or IQGAP1 binding with HGF stimulated in androgen-independent prostate cancer 22, 56. In addition, the kinase activity can also be enhanced by MKK6 and p38 MAPK 57. However, most studies have focused on PAK6 localized in the nucleus and cytoplasm, while the subcellular structures-cell energy centers-PAK6 in the mitochondria have rarely been investigated. In the present study, it was identified by electron microscopy that the overexpression of PAK6 could increase the number of mitochondria in cells. Furthermore, the PAK6 localization in the mitochondrial inner membrane was confirmed by colloid gold labeling through immunoelectron microscopy, proving that PAK6 plays an important role in the mitochondria of tumor cells (Figure 1).

SIRT4 is one of the mitochondrial members of the SIRT family, which has the functions of ADP-ribose transferase and lysine deacylase 26. Early studies have focused on its metabolic function and obesity. Subsequently, this has been reported to have tumor suppressive activity, and regulate the cellular metabolic response to DNA damage by inhibiting mitochondrial glutamine metabolism 34. The low expression of SIRT4 in various tumor tissues has also been reported, which was found to be closely correlated to poor prognosis 32, 34, 58. To date, the clinical impact of SIRT4 on prostate cancer has yet to be defined. In the present study, it was identified that ubiquitination induces the post-translational modification of SIRT4 (Figure 2).

Further studies are required to determine whether SIRT4 is the substrate of PAK6, and the detailed mechanisms, including the identification of the precise ubiquitinated residues and the specific phosphorylation site, and uncover the mechanism of crosstalk between these two modifications. Nevertheless, the results of the present study suggest the strong regulatory relationship between PAK6 and SIRT4.ANT2 is one of the members of the adenine nucleotide transposase family. Its mechanism of action differs from other members of the ANT family. As one of the most abundant proteins expressed in the inner lining of tumor granules, ANT2 maintains the glycolysis-enhanced metabolism of tumors by exchanging ADP and ATP in the cytoplasm and mitochondria, in order to maintain the survival of tumors 36. This has been shown to be closely associated with a variety of malignancies, such as breast cancer, cervical cancer and liver cancer 37, 38. Although ANT2 has been reported to be a regulatory substrate for SIRT4 in literatures 23, 24, the specific mechanism remains not clear. The conclusion of the present study confirms that SIRT4 regulates the acetylation level of ANT2 at K105, and degrades ANT2 by promoting its ubiquitination, which depends on the deacetyation activity of SIRT4 (Figure 3). It has been reported that silencing ANT2 has been shown to enhance the characteristics of apoptosis, and induce apoptosis in human breast cancer cells, thereby inhibiting tumor growth *in vivo* 59. Therefore, it was considered that the degradation of ANT2 by SIRT4 would provide a new target for the treatment of prostate cancer.

In line with these results, the present study supports the notion that PAK6 promotes the ubiquitination degradation of SIRT4, while SIRT4 promotes the ubiquitination degradation of ANT2 by deacetylating ANT2. Furthermore, it has been speculated that PAK6 can affect ANT2 acetylation through the PAK6-SIRT4-ANT2 pathway, thereby stabilizing its protein expression, or that PAK6 directly phosphorylates ANT2 at T107. This also confirms that the interplay of phosphorylation and deacetylation of ANT2 regulates apoptosis of prostate cancer cells.

The growth of prostate cancer cells does not necessarily depend on glucose uptake, but manifests glutamine-dependent growth, which is a phenomenon known as "glutamine addiction" 48, 49. Therefore, glutamine may play a key role in prostate cancer. Similar to glucose metabolism, the increase in uptake and utilization of glutamine by tumor cells is also regulated by oncogenes. For example, c-myc is co-regulated by the glutamine transporter and glutaminase, in order to increase glutamine metabolism. Rho GTPase enhanced the activity of glutamine enzymes, and increased the metabolism of glutamine through a NF-κB dependent approach, promoting tumor progression 60, 61. However, the relationship between PAK6, which is an effector of Cdc42/Rac1 of the Rho GTPase family, and glutamine metabolism has not been reported. These present results show that SIRT4 reverses PAK6-mediated ANT2 acetylation depending on glutamine. However, the regulation of ANT2 acetylation by PAK6 is independent of glutamine (Figure 4), which suggests that a high expression of PAK6 can maintain the protein stability and acetylation of ANT2 in the absence of glutamine, providing conditions for the survival of prostate cancer with the lack of glutamine. Indeed, the specific mechanism remains to be explored.

In the present study, it was demonstrated that ANT2 was phosphorylated by PAK6 at T107. Meanwhile, ANT2 was deacetylated by SIRT4 at K105. Furthermore, it was confirmed that ANT2 acts as a dual substrate for the two enzymes, PAK6 and SIRT4. Since protein phosphorylation and acetylation modification can interact with each other 50, and the predicted ANT2 acetylation at K105 is adjacent to the phosphorylation at T107, it can be speculated that there may be a mutual regulation. These experimental results revealed that the phosphorylation motif of T107 was damaged by the K105 mutant, leading to the change in ANT2 phosphorylation.

Meanwhile, T107A also changed the acetylation of ANT2 (Figure 5), confirming the mutual regulation between ANT2 phosphorylation and acetylation. Previous studies have shown that PAK6, SIRT4 and ANT2 are separately correlated to apoptosis. The present study demonstrates that silencing PAK6 or ANT2 can induce apoptosis in prostate cancer cells. Furthermore, PAK6 manipulates apoptosis by regulating ANT2, in terms of protein stability and its post-translational modifications, including the phosphorylation and acetylation of ANT2. The tumorigenesis experiment in nude mice confirmed this conclusion. Moreover, the clinical sample evaluation also confirmed the correlation of the PAK6-SIRT4-ANT2 complex.

In conclusion, a novel PAK6-SIRT4-ANT2 complex was uncovered in the mitochondria, and ANT2 was identified as a new substrate of PAK6. The de-acetylation of ANT2 to ubiquitization, and the phosphorylation of ANT2 to deacetylation were coupled in the post-translational modification in prostate cancer apoptosis. These present findings provide insights into the new mechanism of PAK6 in the mitochondria, indicating that ANT2 and its phosphorylation and de-acetylation may play a critical role in the apoptosis of prostate cancer. These might open up new potential therapeutic avenues for the treatment of prostate cancer.

## Abbreviations

Ala: Alanine
ANT2: adenine nucleotidetranslocase-2
Ac-ANT2: Acetylationadenine nucleotidetranslocase-2
DAPI: 4',6-diamidino-2-phenylindole
FBS: fetal bovineserum
GFP: Green fluorescenceprotein
GST: Glutathione-s-transferase
IHC: immunohistochemistry
Lys: Lysine
Mit: Mitochondria
PAK6: p21-activated kinase6
PBS: phosphate bufferedsaline
PBST: phosphate buffered saline supplemented with Tween-20
PCR: Polymerase Chain Reaction
PI: protein kinaseinhibitor
Arg: Arginine
Ser: Serine
SIRT4: NAD-dependent protein lipoamidase sirtuin-4
Thr: Threonine
Ub: Ubiquitin
WT: wildtype
